# Potency and breadth of human primary ZIKV immune sera shows that Zika viruses cluster antigenically as a single serotype

**DOI:** 10.1371/journal.pntd.0008006

**Published:** 2020-04-13

**Authors:** Chad D. Nix, Jonathan Salberg, Felicity J. Coulter, Bettie W. Kareko, Zoe L. Lyski, Brian L. Booty, William B. Messer

**Affiliations:** 1 Dept. of Infection Prevention and Control, Oregon Health and Science University, Portland, Oregon, United States of America; 2 Dept. of Molecular Microbiology and Immunology, Oregon Health and Science University, Portland, Oregon, United States of America; 3 Dept. of Medicine, Division of Infectious Diseases, Oregon Health and Science University, Portland, Oregon, United States of America; 4 Oregon Clinical and Translational Research Institute, Oregon Health and Science University, Portland, Oregon, United States of America; 5 Program in Epidemiology, Oregon Health & Science University–Portland State University School of Public Health, Portland, Oregon, United States of America; International Centre for Genetic Engineering and Biotechnology, INDIA

## Abstract

Zika virus (ZIKV) emerged as a global public health threat throughout the Americas since 2014. Phylogenetically, the virus is composed of three main lineages, an African, Asian, and American lineage. The recent emergence and spread of ZIKV has raised questions regarding the breadth and potency of human primary ZIKV immune sera against antigenically diverse ZIKV. Although ZIKV is thought to compose a single antigenic serotype, in-depth evaluation of the antigenic relatedness of ZIKV across genetic variants has been limited to a relatively small series of early convalescent human immune sera (4–12 weeks) against a limited number (3) of genetic variants. Using virus neutralization assays, we characterize the potency and breadth of twelve primary ZIKV immune sera from adults infected 5 to 38 months previously against a panel of 11 ZIKV isolates from the African, Asian and American lineages. We assess the variability of neutralization potency of immune sera from these subjects and the variability of susceptibility to neutralization for each virus isolate. Overall, we found all sera neutralized all viruses at FRNT_50_ ranging from 1:271 to 1:4271, a 15.8-fold range, with only small differences between subject geometric mean titers (GMT) against all viruses and small differences between each ZIKV isolate and sensitivity to neutralization by all sera: when pooled, African strains were 1.3-fold more sensitive to neutralization by subject immune sera compared to pooled American strains. Finally, we subjected our data to analysis using antigenic cartography, finding that ZIKV are highly antigenically similar, with only a ~4-fold range across all antigenic distances between viruses, consistent with a single serotype.

## Author summary

The recent emergence of Zika virus as an important human pathogen has raised questions about the durability and breadth of Zika virus immunity following natural infection in humans. While global epidemic patterns suggest that Zika infection elicits a protective immune response that is likely to offer long-term protection against repeat infection by other Zika viruses, only one study to date has formally examined the ability of human Zika immune sera to neutralize different Zika viruses. That study was limited because it evaluated human immune sera no more than 13 weeks after Zika virus infection and tested a relatively small number of Zika viruses. In this study, we examine twelve human Zika immune sera as far as 3 years after infection and test the sera against a total of eleven Zika virus isolates. Our results confirm the earlier study and epidemic patterns that suggest Zika virus exists in nature as a single serotype, and infection with one Zika virus can be expected to elicit protective immunity against repeat infection by any Zika virus for years to decades after the first infection.

## Introduction

Although Zika virus (ZIKV) was first isolated in 1947 from a rhesus monkey in the Zika Forest of Uganda[1, 2], only in the last decade has ZIKV emerged as a global public health threat. The first large outbreak among humans was characterized by an epidemic of fever and rash on the Island of Yap in 2007.[3, 4] ZIKV then went largely quiescent until introduction to French Polynesia in 2013, where an epidemiologic association with Guillain-Barré syndrome (GBS) was identified.[5] A rapid expansion ensued as ZIKV spread throughout Oceania, progressing to the Americas in 2015.[6] Concern for the consequences of ZIKV infection heightened as an increased number of infants born with microcephaly were recorded in areas of confirmed ZIKV transmission in Brazil. The association between congenital abnormalities and GBS with ZIKV infection prompted the World Health Organization (WHO) to declare a Public Health Emergency of International Concern on February 1, 2016 (PHEIC).[7] Although ZIKV remains a significant long-term public health challenge, on November 18, 2016, the WHO announced that the ZIKV epidemic no longer met criteria for representing a PHEIC as the number of reported cases decreased.

ZIKV is a single-stranded positive-sense RNA of the *Flavivirus* genus transmitted by *Aedes spp*. mosquitoes. It has historically been assigned two major lineages, an African lineage and an Asian lineage.[8] However, recent analyses suggest a third, American lineage, has emerged from the Asian lineage.[9] The ZIKV virion contains three structural proteins: capsid (C), pre-membrane/membrane(prM/M), and the envelope (E) glycoprotein. The E glycoprotein is the primary target of virus-neutralizing antibodies following natural infection, and these antibodies are thought to be primarily responsible for long-term protection against repeat infection.[9] ZIKV E glycoproteins are highly conserved, with amino acid divergence at ~6% between ZIKV lineages and ~2% within lineages. Phylogenetic analyses suggest that ZIKV exists as a single serotype, however, significant differences in neutralization titers in dengue virus (DENV) have been explained by as few as two amino acid substitutions,[10] and the amount of amino acid variability needed to produce a distinct DENV serotype is difficult to predict.[11]

Historically, immune sera raised in humans, mice, or non-human primates (NHP) have been used to formally characterize flavivirus serotypes, with the first two DENV serotypes being defined using five viruses isolates and four human convalescent immune sera[12]. What we now know of as the four DENV serotypes were first defined using fourteen clinical virus isolates, four NHP sera and an unreported number of mouse sera.[13] The original characterization of ZIKV as a distinct virus used a single ZIKV isolate and ZIKV immune sera from a single NHP and sera from fourteen NHPs infected with other known sylvatic viruses [2]. However, the correlation between serotype-specific antibodies raised in experimentally infected animals and human serotype-specific immunity following natural infection has never been established. To more fully characterize the potency and breadth of human ZIKV immune sera many months to years after primary (single flavivirus) natural infection and empirically evaluate the antigenic relationship between ZIKV, we tested a panel of twelve human sera collected 5 to 38 months following ZIKV infection against eleven genetically distinct ZIKV isolates. Immune sera potency and breadth were assessed using 50% focus reduction neutralization tests (FRNT_50_) and we evaluated antigenic relatedness using antigenic cartography. These study results confirm and advance the early work by others and have important implications for ZIKV vaccine design and evaluation. Additionally they provide a solid basis for estimating and understanding the very limited potential for antigenically variant ZIKV viruses to penetrate otherwise ZIKV immune populations.

## Methods

### Human research ethics

The study has been reviewed and approved by the Oregon Health & Science University Institutional Review Board (IRB#10212). Informed consent was obtained from subjects on initiation of their participation in the study.

### Study population

ZIKV immune individuals in this study were enrolled in a larger study of long-term immunity following infection with the arthropod-borne DENVs, ZIKV, and chikungunya virus (CHIKV). Prospective ZIKV study subjects were initially identified with the assistance of the Oregon Health Authority (OHA) to whom the CDC reports PCR or serologically confirmed ZIKV cases among Oregon residents. These individuals were invited to participate in the study via US Postal Service mail. Subjects who contacted the long-term immunity study were offered participation in the study, and following informed consent, provided additional history including other known and suspected arboviral infections, lifetime travel histories, and yellow fever virus (YFV) and Japanese encephalitis virus (JEV) vaccination histories.

### Sample collection and storage

On enrollment, subjects provided approximately 80 mL of blood, with 30 mL collected in BD “red top” serum separator vacutainers (Becton-Dickson) for serologic studies and stored at -80°C until used for assays.

### Viruses and tissue culture

All ZIKVs and yellow fever virus strain 17D (YFVax®) were propagated in C6/36 mosquito cells (ATCC CRL 1660) in minimal essential media (MEM) supplemented with L-glutamine (Gibco), non-essential amino acids (NEAA) (Gibco), antibiotic antimycotic (anti-anti) (Gibco) and 5% by volume fetal bovine serum (FBS) incubated at 32°C, 5% CO_2_. Zika viruses included PRVABC59 (Puerto_Rico_2015) and FSS13025 (Cambodia_2010), both generously provided by the World Reference Center for Emerging Viruses and Arboviruses (WRCEVA), and ZIKV/Homo sapiens/COL/FLR/2015 (Colombia_2015), BeH819015 (Brazil_2015), MEX 2–81 (Mexico_2016), 41525 (Senegal_1984), IbH_30656 (Nigeria_1968), and H/PF/2013 (French_Polynesia_2013), generously provided by Alec Hirsch, PhD, OHSU Vaccine and Gene Therapy Institute. Strains MR766 (Uganda_1947), P 6–740 (Malaysia_1966), and PLCal_ZV (Thailand_2013) were obtained from BEI resources. 17D was obtained from the manufacturer (Sanofi USA). All DENV were propagated in Vero cells that over-express furn. [14, 15] VF cells were grown in MEM with 10% FBS, NEAA, anti-anti, and selection antibiotic G418 (InvivoGen) at 37°C and 5% CO_2_ DENV used in neutralization assays included infectious clones of DENV3 UNC3001,[16] and DENV4 DV4SL1992a[17] as well as DENV1 WestPac’74 (generously provided by Stephen Whitehead, National Institutes of Health) and DENV2 16803 (WRCEVA). All neutralization assays were performed using Vero cells (ATCC CCL-81) similarly grown with MEM, NEAA, anti-anti and 10% by volume FBS incubated at 37°C and 5% CO_2_.

### Neutralization assays

Fifty percent focus reduction neutralization test (FRNT_50_) titers were used to characterize subject sera. Subject sera were first heat-inactivated at 56°C for 30 minutes. Sera were then diluted four-fold in MEM supplemented with 2% FBS from a starting dilution of 1:10 and mixed with an equal volume of ~100 focus forming units (FFU) of ZIKV strains giving a final starting dilution of 1:20. Virus-dilution mixes without sera were prepared simultaneously as controls for input virus FFUs. After 1 hour of incubation, virus mixes were inoculated into individual wells of 96 well plates seeded with Vero cells, incubated for 1 hour, and overlaid with 1% methylcellulose in Opti-MEM (Gibco) supplemented with NEAA, anti-anti, amphotericin B, and 2% FBS. Plates were incubated for 24 hours at 37°C, 5% CO_2_. The overlay was then removed, monolayers were fixed with 4% formaldehyde, incubated with the anti-flavivirus mouse monoclonal antibody (4G2) (ATCC HB 112) followed by a horse-radish peroxidase (HRP) conjugated secondary goat-anti-mouse antibody (ThermoFisher Scientific Cat # 62–6520). Plates were washed 3 times with PBS and HRP True Blue Peroxidase Substrate (KPL) was then applied for 15–60 minutes to visualize infected foci. Using a CTL ImmunoSpot instrument (CTL, Cleveland, OH, USA), foci in individual wells were scanned, counted, and counts underwent quality control. Proportion of virus neutralized per well was calculated and the serum dilution that neutralizes 50% of control input virus (FRNT_50_) was determined by sigmoidal dose-response curve fitting of percent neutralization vs. fold serum dilution using GraphPad Prism® (Version 7.0). All assays were performed in biologic duplicates. Individual sera that showed a greater than 4-fold difference between biologic replicates were subjected to repeat neutralization assays. Initial neutralization assays against DENV1-4 and YFV 17D were conducted in a similar manner in a 24-well plate format with 30–40 PFU/well. DENV plates were incubated for 5 days and counter stained for infectious foci in the same manner as ZIKVs, 17D plates were incubated for 7 days and counter stained with crystal violet to visualize infectious plaques. Fifty-percent neutralization titers for all assays are reported as the fold-dilutions.

#### Antigenic cartography

The ZIKV antigenic map was constructed as previously described[11, 18] and implemented using the Acmacs Web Cherry platform (https://acmacs-web.antigenic-cartography.org/). Briefly, antigenic maps are constructed by first generating a table of antigenic distances (*D*_*ij*_) between each individual virus (*i*) and serum (*j*) using serum titers for each serum-titer pair (*N*_*ij*_). To calculate table distance, the titer against the best neutralized virus for that serum is defined as *b*_*i*_ and the distances for that serum are calculated as *D*_*ij*_ *= log*_*2*_*(b*_*i*_*)-log(N*_*ij*_*)*. For the best neutralized virus for that serum, *N*_*ij*_ *= b*_*i*_, and this distance will be equal to 0. For the remaining serum-virus pairs, table distance *D*_*ij*_ is equivalent to the fold-difference in titer between *b*_*ij*_ and *N*_*ij*_. Euclidean map distance (*d*_*ij*_) for each serum-virus pair is found by minimizing the error between the table distance D_ij_ and map distance, d_ij_, using the error function *E = ∑*_*ij*_*e(D*_*ij*_,*d*_*ij*_*)*, where *e(D*_*ij*_,*d*_*ij*_*) = (D*_*ij*_*-d*_*ij*_*)*^*2*^ when the neutralization titer is above 1:20. For viruses with neutralization titers <1:20, the error was defined as *e(D*_*ij*_,*d*_*ij*_*) = (D*_*ij*_*-1-d*_*ij*_*)*^*2*^*(1/1+e*^*-10(Dij-1-dij)*^*)*. To make a map and derive *d*_*ij*_ for each serum-virus pair, viruses and sera are assigned random starting coordinates and the error function is minimized using the conjugate gradient optimization method. One thousand independent optimizations were conducted to generate the aintgenic map.

### Statistical analyses

The evolutionary history for the genetic relatedness of ZIKV was inferred by using the Maximum Likelihood method and General Time Reversible model. The tree with the highest log likelihood was retained. Initial tree(s) for the heuristic search were obtained automatically by applying Neighbor-Join and BioNJ algorithms to a matrix of pairwise distances estimated using the Maximum Composite Likelihood (MCL) approach, and then selecting the topology with superior log likelihood value. Codon positions included were 1st+2nd+3rd. There were a total of 2382 positions in the final dataset. Evolutionary analyses were conducted in MEGA X.[19]

Geometric mean titers between sera were plotted and compared in GraphPad Prism (Version 8.00 for Mac, GraphPad Software, La Jolla California USA, www.graphpad.com) using ANOVA followed by Bonferroni’s correction for multiple comparisons. Potency and breadth survival curves were assembled in GraphPad Prism and compared using a Mantel-Cox non-parametric comparison followed by Bonferroni’s correction for multiple comparisons (adjusted P = 0.05). Correlation between geometric mean titer (GMT) or area under the curve (AUC) for survival curves and months post-infection was assessed using standard least-squares fitting of log_10_ transformed FRNT_50_ or AUC and months post-infection (power model) using JMP® (Version 14.0.0. SAS Institute Inc., Cary, NC, 1989–2019.)

### Sequencing

Viral RNA was isolated from tissue culture supernatants using QIAamp Viral RNA Mini Kit (Qiagen). cDNA was generated using random hexamers and SuperScript II Reverse Transcriptase (Thermo Fisher), according to the manufacturer’s instructions. Capsid genes were amplified from cDNA using primers ZV_seqA_s and ZV_seqA_a using Phusion Polymerase according to the manufacturer’s instructions. Fifty μL reactions (1X HF buffer (Thermo Fisher), 200mM each dNTP, 0.5mM forward primer, 0.5mM reverse primer, 1U Phusion Polymerase and 4 mL cDNA) were subjected to thermocycling conditions of 98°C for 30 seconds, followed by 40 cycles of 98°C for 10 seconds, 58°C for 15 seconds and 72°C for 15 seconds, prior to final extension at 72°C for 10 minutes. prM, and E genes were amplified from cDNA using primers ZV_seqB_s and ZVseqF_a and Phusion Polymerase as 50 μL reactions as before (S1 Table). Thermocycling conditions consisted of 98°C for 30 seconds, followed by 40 cycles of 98°C for 10 seconds, 55°C for 15 seconds and 72°C for 80 seconds, prior to final extension at 72°C for 10 minutes. PCR products were visualized on 1.5% agarose gel with ethidium bromide, purified (QIAquick PCR Purification Kit, Qiagen) and mixed with sequencing primers (S1 Table) for submission to a commercial sequencing company (Genewiz) for Sanger sequencing. Contigs were assembled from chromatographs and aligned using Geneious Prime (version 2019.2.1).

## Results

### Study subjects

A total of 12 subjects with history of ZIKV infection (Table 1) and without serologic evidence of DENV infection (Table 2) were identified. Clinical infections were documented between February 2014 and December 2016 and individual subject sera were obtained anywhere from 5 to 38 months post-infection. Infections were primarily acquired in Latin America or the Caribbean with two infections documented in the Cook Islands, five subjects had documented yellow fever vaccinations, all of whom had detectable YFV neutralizing antibodies (Table 2).

**Table 1:** Study subjects.

ID	Sex	Age	Country of infection	Months post-infection	Year received yellow fever vaccine
11884	Male	28	Nicaragua	5	NV
14269	Female	45	Cuba	10	NV
14278	Female	51	Mexico	10	NV
14276	Female	52	Virgin Islands	12	NV
14252	Female	24	Mexico	13	2013
14664	Female	39	Jamaica	14	2002
11981	Female	44	Dominican Republic	15	2008
14236	Male	55	Nicaragua	15	1997
14137	Female	41	Guatemala	18	2003
12451	Female	61	Cook Islands	24	NV
12462	Male	69	Cook Islands	27	NV
11942	Female	25	Haiti	38	NV

NV = Not Vaccinated

**Table 2:** Subject neutralization profiles by fold serum dilution yielding 50% neutralization (FRNT50).

ID	ZIKV	DENV1	DENV2	DENV3	DENV4	YFV
11884	586	<20	<20	<20	<20	<20
14269	1480	<20	<20	<20	<20	<20
14278	1037	<20	<20	<20	<20	<20
14276	1046	<20	<20	<20	<20	<20
14252	2120	<20	<20	<20	<20	159
14664	1164	<20	<20	<20	<20	695
11981	1409	<20	<20	<20	<20	22
14236	2108	<20	<20	<20	<20	180
14137	637	<20	<20	<20	<20	378
12451	895	<20	<20	<20	<20	<20
12462	932	<20	<20	<20	<20	<20
11942	610	<20	<20	<20	<20	<20

ZIKV = Peurto_Rico_2015

#### Zika viruses

**Fig 1:**
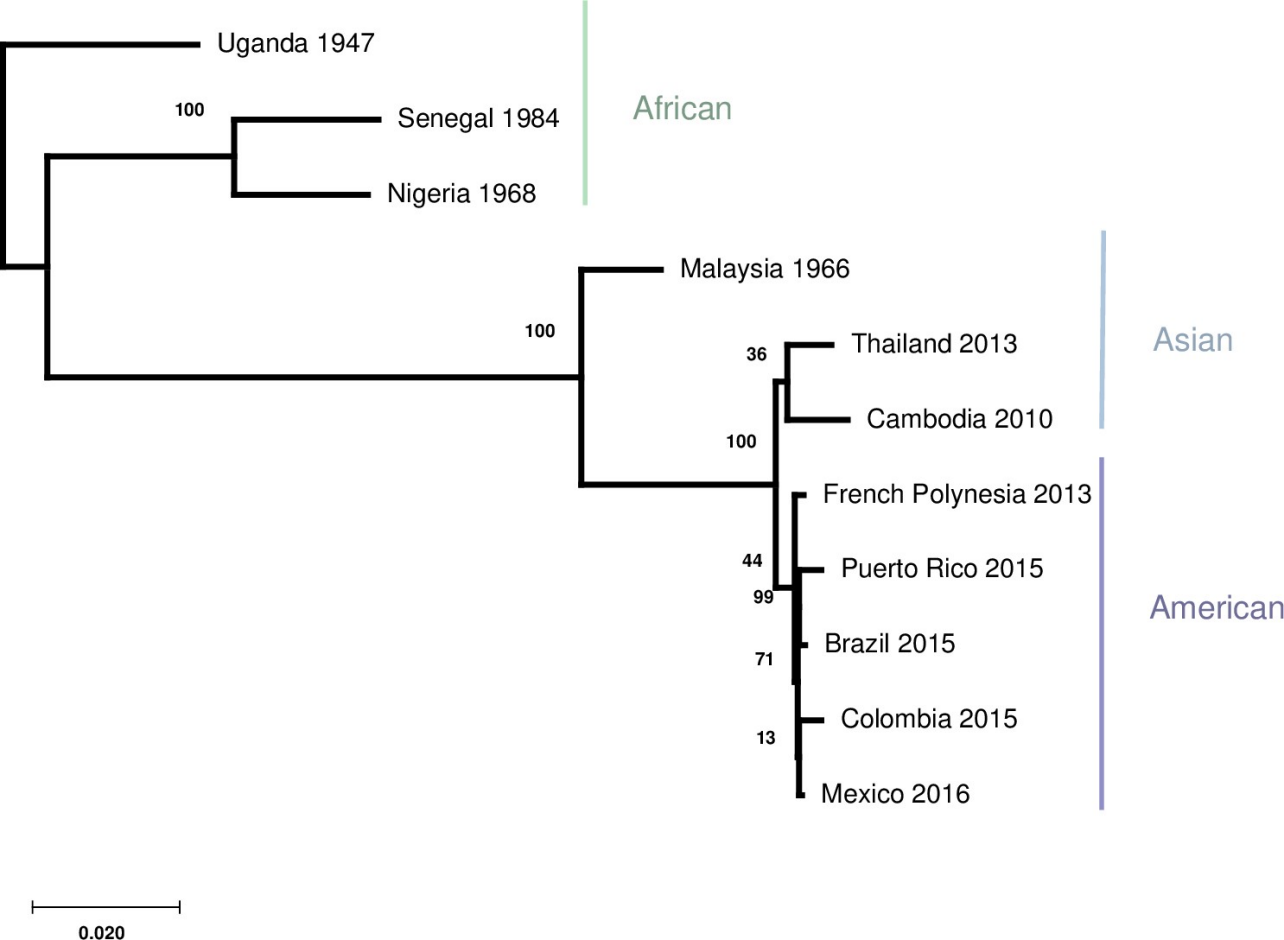
Molecular phylogenetic analysis by maximum likelihood method. The percentage of trees in which the associated taxa clustered together is shown next to the branches. The tree is drawn to scale, with branch lengths measured in the number of substitutions per site. Colored bars correspond to virus strain region of isolation.

ZIKV immune sera from the 12 subjects were tested against eleven ZIKV strains (Table 3): five of American lineage, three of Asian lineage, and three of African lineage. Overall amino acid homology in the ZIKV structural proteins C/prM/E between these strains range from 95.7–99.7% (mean: 98.2%). The viruses grouped by area of geographic isolation with the French Polynesian isolate most closely related to the four Latin American isolates (Fig 1).

**Table 3:** 

Virus strain name	Isolate	Accession #	Year isolated	Location
Uganda_1947	MR766	MN755624	1947	Uganda
Senegal_1984	41525	MN755616	1984	Senegal
Nigeria_1968	IbH_30656	MN755617	1968	Nigeria
Malaysia_1966	P 6–740	MN755625	1966	Malaysia
Cambodia_2010	FSS13025	MN755621	2010	Cambodia
Thailand_2013	PLCal_ZV	MN755626	2013	Thailand
French_Polynesia_2013	H/PF/2013	MN755619	2013	French Polynesia
Puerto_Rico_2015	PRVABC59	MN755623	2015	Puerto Rico
Brazil_2015	BeH819015	MN755622	2015	Brazil
Colombia_2015	ZIKV/Homo sapiens/COL/FLR/2015	MN755620	2015	Colombia
Mexico_2016	MEX 2–81	MN755618	2016	Mexico

#### Virus specific neutralization by subject

**Fig 2:**
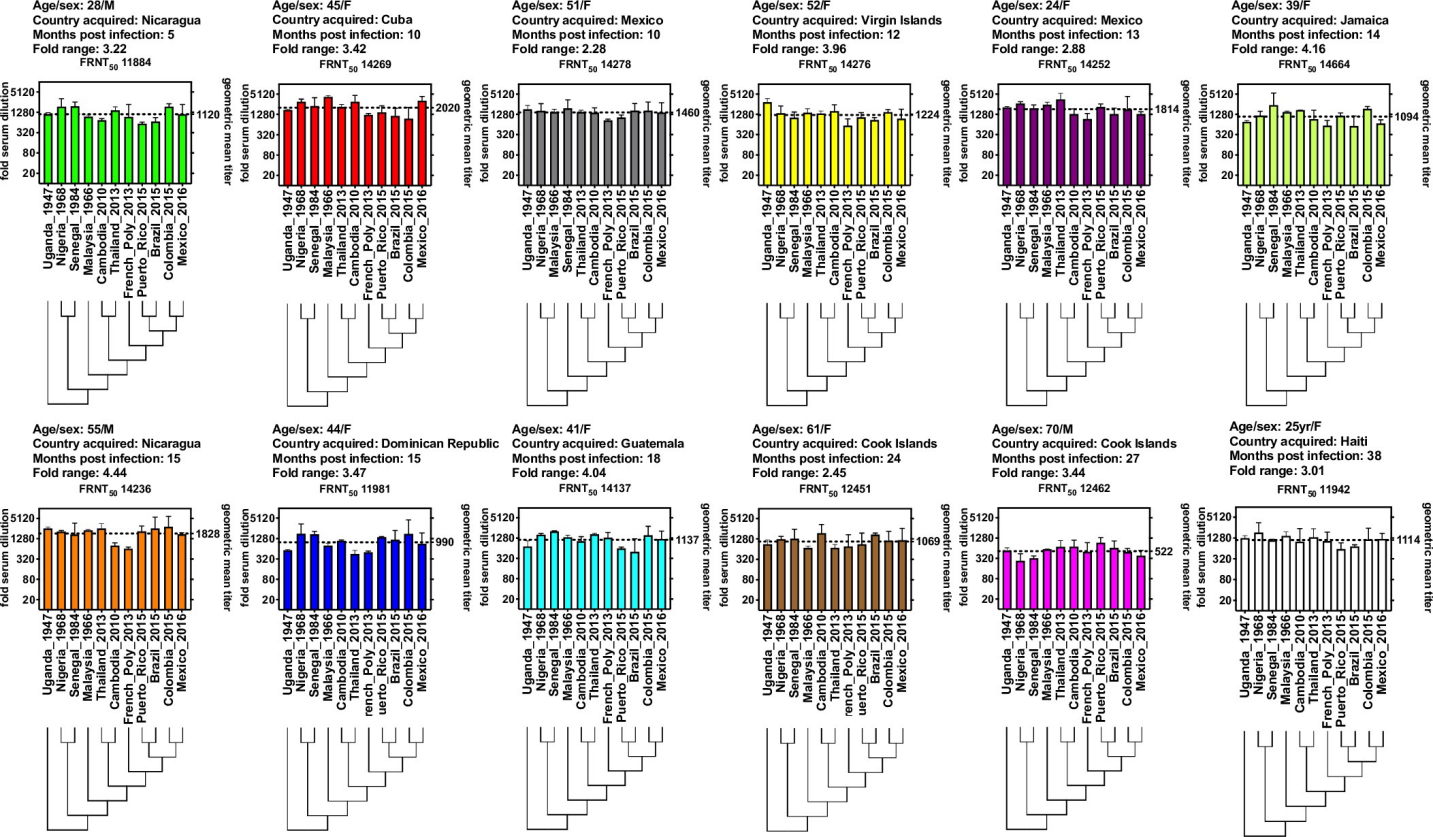
FRNT_50_ titers for subject sera against each virus. Fold serum dilution is shown on the left Y-axis, geometric mean titer (GMT) for each subject against all 11 viruses is plotted on the right Y-axis. Viruses and their relative phylogenetic relationships are shown on the X-axis. Subject age, gender, country infection acquired, months post-infection and fold-FRNT_50_ range are reported above each serum plot. The sera are ordered left to right from most recent to most remote infection.

We first evaluated individual serum neutralizing potency against each of the eleven viruses (Fig 2). Individual FRNT_50_ values between sera ranged from a serum dilution of 1:271 (serum 12462 vs Senegal_1968) to a serum dilution of 1:4271 (serum 14269 vs Malaysia_1966), a 15.8-fold range between all subjects and all viruses. However, within subject variability was substantially less, ranging from 2.28-fold (subject 14278) to 4.44-fold (subject 14236) (Fig 2). None of the titers showed significant differences within subjects (ANOVA).

#### Between subject serum potency and breadth

**Fig 3:**
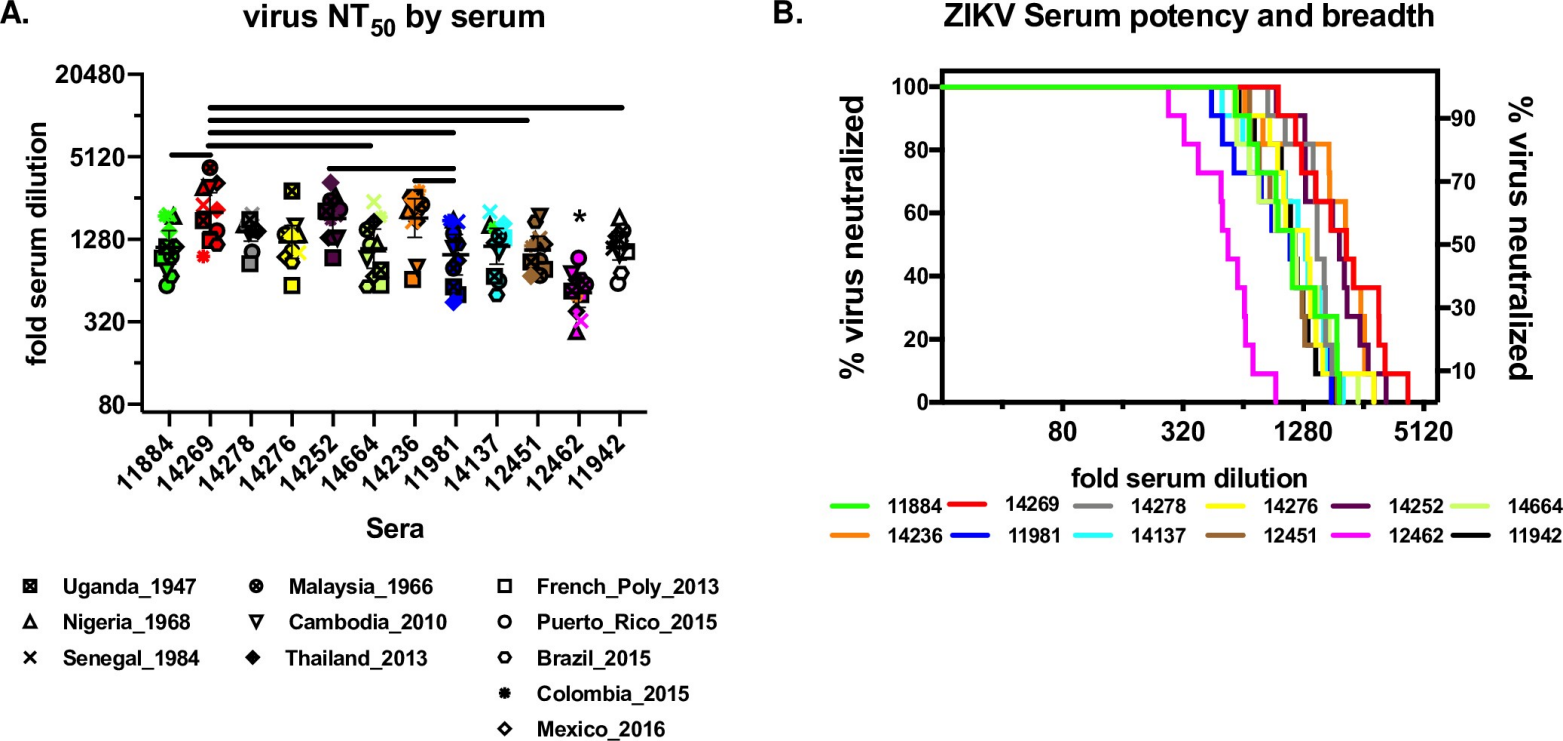
Potency and breadth of subject immune sera. A) GMT for each serum against eleven ZIKV isolates. Y-axis shows fold-serum dilution, each virus FNRT_50_ value is plotted by the correlating symbol show in the legend below the plot. The symbols are color coded to match the sera indicated in Fig 2. Line and whiskers show serum GMT and 95% confidence. The GMT for subject 12462 (*) was significantly lower than all other sera (ANOVA followed by Tukey’s correction for multiple comparisons, P<0.05). Remaining sera pairs that had GMTs that differed significantly are show by horizontal bars (ANOVA followed by Tukey’s correction for multiple comparisons, P<0.05) B) Potency and breadth curve for each subject serum. The Y-axes shows proportion of viruses neutralized (N = 11), X axis shows fold serum dilution. The plots are color coded to match the sera in Fig 2. The plot for each serum shows the proportion of viruses neutralized by FRNT_50_ at a given serum dilution. Each step in each plot represents an FRNT_50_ value for one virus; as fold serum dilution increases for each serum, the proportion of viruses neutralized decreases by 1/11 (9.1%) as each FRNT_50_ dilution for each virus is crossed. *differs from 12462, Mantel-Cox followed by Bonferroni’s correction for multiple comparisons, P<0.05.

We next compared geometric mean titers between each subject (Fig 3A) by ANOVA and overall potency and breadth by survival curve analysis (Fig 3B). Overall, individual subject GMT against all eleven viruses had a relatively narrow range, from a low of 1:521(subject 12462) to a high of 1:2020 (subject 14269), or a 3.9-fold difference. Subject 12462 serum GMT was significantly lower than all other subject sera, subject 11981 had a significantly lower GMT (1:990) against 4 subject sera, and subject 14269 had a significantly higher GMT against 5 other subject sera (ANOVA followed by Tukey HSD, Fig 3A). As an alternate approach to visualizing and comparing individual subject neutralization profiles, we constructed potency and breadth curves for each subject (Fig 3B). Potency and breadth neutralization curves were first developed in the HIV field to characterize broadly neutralizing antibodies and immune sera against panels of HIV pseudoviruses (de Camp et al, 2014). These curves maintain resolution at the level of neutralization titer against each virus, rather than calculating a mean estimate based on all titers. Curves show the number of viruses neutralized at a given serum dilution over the range of all dilutions tested, analogous to survival over time in a Kaplan-Meier curve. Curves are compared using the non-parametric Mantel-Cox test followed by a Bonferroni correction for multiple comparisons. This approach does not assume a normal distribution of individual titers, but is also less sensitive to differences between curves. Upon inspection, 12462 again stands out as different from all other subjects’ neutralization pattern, although the difference was only statistically significant for sera 14278, 14252, 14236, 11981, 12451 and 11942. None of the other eleven sera tested differed from one-another in potency or breadth. Finally, we tested whether there was a correlation between GMT (Fig 3A) or area under the curve (Fig 3B) and months post-infection, finding no correlation between time since infection and subsequent ZIKV neutralizing antibody titers (adjusted R^2^ = -0.1, P = 0.80 for both GMT and AUC).

#### Neutralization by virus and antigenic cartography

**Fig 4:**
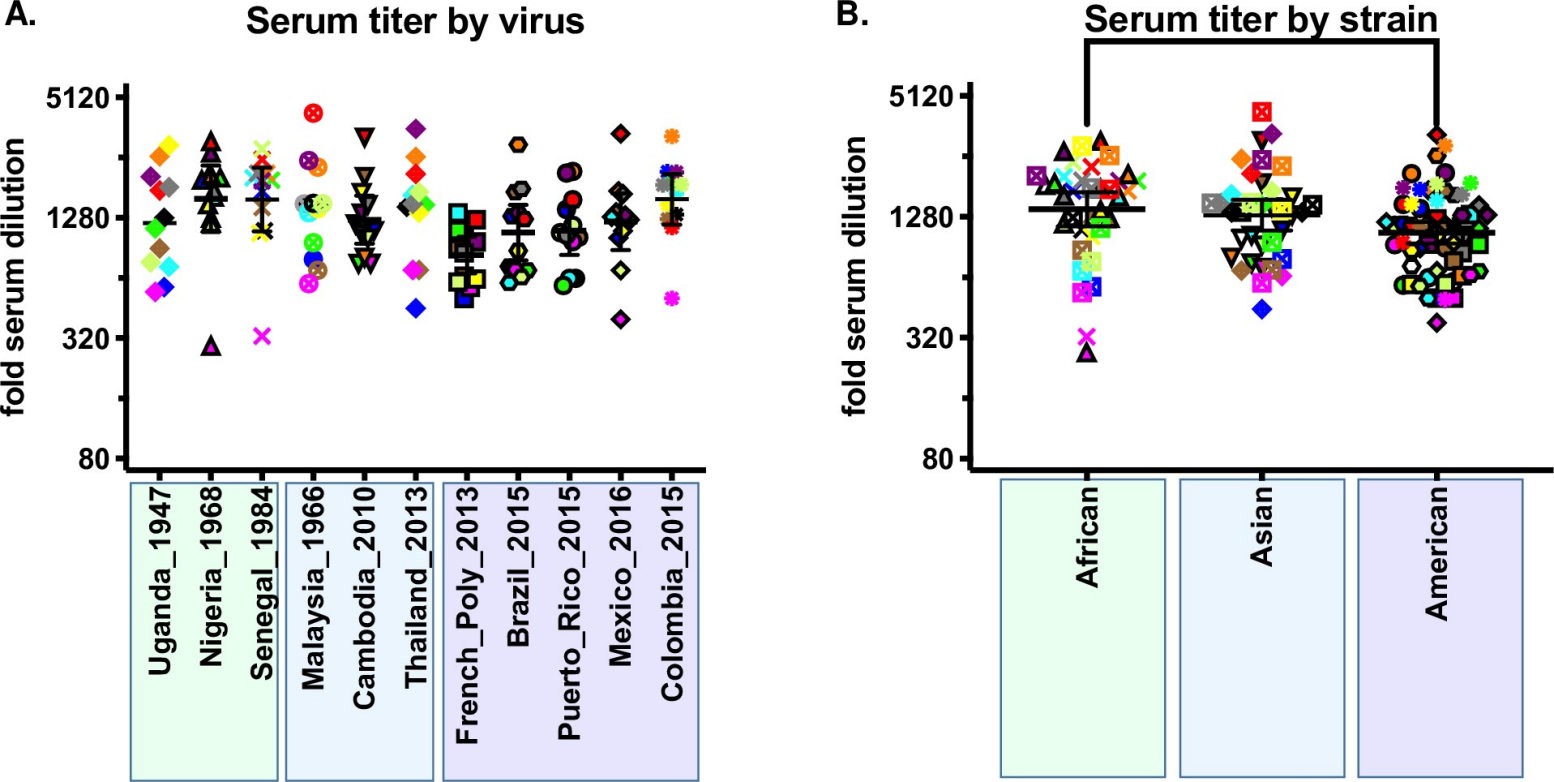
Neutralization by virus and strain. A) FRNT_50_ titers for each serum against each virus. The Y-axis shows fold-serum dilution, the X-axis shows each virus, the phylogenetic tree below the X-axis shows the relative genetic relatedness of the viruses. Individual virus symbols are indicated below the figure and the symbols are color-coded to match the sera in Fig 2. B) FRNT_50_ titers for ZIKV grouped by strain. Strain pairs found to be different by ANOVA followed by Tukey HSD correction for multiple comparisons are show by brackets (P = 0.038).

To specifically address the relative differences in virus sensitivity to neutralization by human immune sera, we first compared GMTs for each virus against all sera (Fig 4A). GMTs for each virus ranged from 1:836 for French_Poly_2013 to 1:1594 for Nigeria_1968, a 1.91-fold difference. None of the differences between any of the GMTs by virus were statistically significant (ANOVA followed by Tukey HSD post-hoc test). When comparted by strain, the GMTs for African, Asian, and American were 1:1400, 1:1306, and 1:1067, respectively, with the African and the American strains differing significantly from one another, a 1.31-fold difference (ANOVA followed by Bonferroni, P = 0.016).

**Fig 5:**
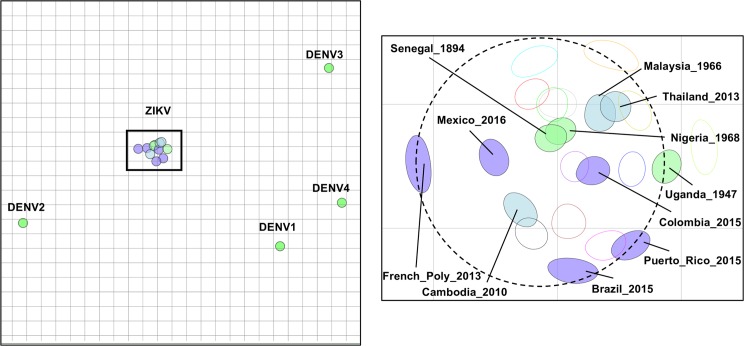
Antigenic map of eleven ZIKVs against twelve human ZIKV immune sera from 5 to 38 months post-infection. Locator map shows the relative antigenic relatedness between ZIKV and the dengue viruses. Each unit of antigenic distance (AU), the length of one side of a grid square, is equivalent to a two-fold dilution in the neutralization assay. Inset map shows each serum and ZIKV. Sera are shown as open blobs colored to match each serum as in Fig 2. Each virus is shown as a filled blob and is colored according to virus strain **(American = slate blue, African = turquoise, Asian = steel blue**). The size and shape of each blob is the confidence area of its position. Dashed line circle has a diameter equal to 2 AU or four-fold, the difference of a single dilution in the FRNT assay used to estimate FRNT_50_ titer.

To further characterize the antigenic relationship between genetically distinct ZIKV, we turned to antigenic cartography.[11, 18] Antigenic cartography has been implemented previously to describe the antigenic relatedness of dengue viruses[11, 20] and influenza.[18, 21–23] Antigenic maps offer an alternate way to evaluate neutralization titers in that they are based on neutralization data that reflect the antigenic rather than genetic relatedness of pathogens. Viral antigenic maps fit neutralization titers for all sera against all viruses simultaneously: each virus is measured in relation to many immune sera, allowing for a more accurate estimate of antigenic relatedness than that which is provided by individual neutralization titers. Antigenically, ZIKV formed a discrete and compact map, with the greatest antigenic unit (AU) distances no greater than ~four-fold range across the two-dimensional map space (Fig 5), equivalent to a single dilution in the FRNT assay used to estimate FRNT_50_ values for this map. The two African strains mapped to nearly identical loci, while the American strains were located at relatively larger, but still quite small, antigenic distances from one another, while the lone Asian strain in our panel was centrally located among the American and African strains. Compared to the dengue viruses, ZIKV were more tightly clustered and more closely related antigenically than DENV viruses are when mapped using human primary DENV immune sera.[11]

## Discussion

Phylogenetic comparisons between historic and contemporary ZIKV isolates find minimal genetic variability, supporting the generally held hypothesis that ZIKVs antigenically form a single serotype.[9] However, within flaviviruses it has been shown that relatively few genetic—amino acid encoding—differences can be associated with significant differences in virus susceptibility to neutralization by human immune sera raised against otherwise genetically very similar viruses.[10, 16, 24] Consequently, the antigenic differences between closely related flaviviruses cannot be inferred by genetic analysis alone, and empiric characterization of closely related viruses using serologic assays is still necessary to validate assumptions about antigen relationships within genetically similar flaviviruses. Ideally, this characterization should be undertaken with a set of viruses that capture within virus genetic diversity and a set of sera from individuals with diverse exposure histories to the same group of genetically similar viruses. To date, only one study, Dowd *et al*.,[25] has examined the serotype specificity of contemporary human ZIKV convalescent immune sera from the most recent 2014–2016 epidemic against the three genetically distinct ZIKV strains. While Dowd *et al*. was a critically important first approximation of the potency and breadth of human ZIKV-specific immune sera following natural infection, the study had several limitations: only three ZIKV strains were fully evaluated against eight ZIKV-confirmed human convalescent sera; the convalescent sera were collected in an early convalescent time period 3 to 12.6 weeks post infection, when short-lived flavivirus cross-reactive IgM and IgG titers are expected to be high,[26, 27] and, finally, subject co-existent flavivirus immunity to DENV or other flaviviruses in the human subjects was not assessed. Here we conducted an in-depth analysis of a panel of genetically similar but distinct ZIKV against a panel of primary ZIKV-immune sera from individuals infected 5–38 months prior these immunologic studies. Our work builds upon and expands earlier results reported by Dowd *et al*.[25] which was limited to eight early convalescent ZIKV immune sera (<3 months) and only three ZIKV isolates. All subject sera in our study potently neutralized all ZIKV isolates at FRNT_50_ >1:320 (Fig 2), and although the GMT against all viruses for one subject, 12462, was significantly lower compared to the GMTs for the other eleven subjects (Fig 3A), this may reflect a host factor such as subject age at infection– 70 years old–rather than an underlying immunogenic difference between the infecting viruses. The identity of the infecting virus is not known, but can be reasonably assumed to be an American strain (infected in the Cook Islands in 2014), and another subject, 18451, was infected at the same time and location as subject 12462, presumably with the same virus strain, but did not develop lower immune titers. We did not observe any trends towards a relationship between time post-infection, geographic location of infection, and specificity, breadth, or potency of immune sera titers.

When FRNT_50_ titers were compared by virus (Fig 4A), none of the viruses differed in their GMT against the twelve subject sera. However, it is interesting to note that, when pooled by strain, we did find a statistically significant, but small, 1.4-fold difference between GMT for both African strains compared to the five American strains. These two strains have 8 conserved amino acid differences across the E glycoprotein (Supplemental Table), four of which are predicted to be surface-exposed: T120A, located on the E domain II (EDII) d-e loop; V169I, located on the EDI F_0_ strand; V317I, located on the EDIII A strand; and D393E, located on the EDIII f-g loop, part of the EDIII lateral ridge epitope region. While it is beyond the scope of this research to evaluate whether these residues contribute to the small but robust differences in neutralization between the African and American ZIKV strains, all four regions have been implicated as targets for human ZIKV [28] and DENV neutralizing mAbs.[29–31] Even so, the overall magnitude of neutralization potency across all strains and sera suggests these differences are unlikely to play a meaningful role in antigenic escape in the context of existing primary ZIKV immunity.

Analysis of our data by antigenic cartography provided similar results, with some notable differences. While comparison of GMTs across titers and sera suggested some differences between sera and titers in pairwise comparisons, when plotted using all serum-virus pairs simultaneously, the resulting antigenic map is remarkably tight and homogeneous, with neither African or American strains grouping apart from the other strains, and while two African strains were the closest to one another by antigenic distance, the antigenic map distances between the African strains and the American strains are no greater than the antigenic map distances with the American strains. Overall, the antigenic distance spanned by all viruses was just over 2 AU, or four-fold, the difference of a single dilution in our FRNT assay, strongly supporting the hypothesis that ZIKV constitutes a single serotype.

Although our results add significantly to our understanding of the potency and breadth of antibodies elicited by primary ZIKV infection and the antigenic relationship between genetically distinct ZIKV, our study had several weaknesses: the virus strain(s) that infected our study subjects has been inferred to be American strains by epidemic association rather than virus isolation, and subjects with history of primary ZIKV infection by African or Asia strains were not identified; our panel of viruses was biased towards recent American strain isolates, with only three Asian and three African strains represented; and, the study is cross-sectional and still only examines potency and breadth of immune sera through 3 years post-infection, true “long-term” immunity, and longitudinal ZIKV immunity in study subjects was not examined. Future studies that address these weaknesses would substantially add to our understanding of the natural history of ZIKV immunity in human populations, the limited role antibody neutralization escape may play in ZIKV evolution, and the potential for ZIKV vaccines currently under development to protect against likely future genetic ZIKV variants.

## Supporting information

S1 Table Primers used for ZIKV sequencing. (PDF)

S2 Table Raw P-values for pair wise comparisons of potency/breadth curves (Fig 3B). Cells highlighted in red show serum pairs that differ by P<0.05 by Bonferroni correction.
(PDF)

S3 Table Summary of variable residues across ZIKV capsid (C), prM, and envelope (E) proteins. (PDF)
