# Supplementary material for: Potency and breadth of human primary ZIKV immune sera shows that Zika viruses cluster antigenically as a single serotype
Source: PLoS Negl Trop Dis. 2020 Apr 13;14(4):e0008006. doi: 10.1371/journal.pntd.0008006 (PMC7213746; doi:10.1371/journal.pntd.0008006)
Supplement: S1 Table — (PDF) [file pntd.0008006.s001.pdf]

Supplemental Table 1. primers used for ZIKV sequencing.

Name	5' - Sequence - 3'
ZV_seqA_s	ACAGTTCGAGTTTGAAGCG
ZV_seqA_a	TGTRGTCAGCAGGAGGC
ZV_seqB_s	TAGGAAGGAGARGAAGAGACG
ZV_seqB_a	TGTCAAGGTAGGCTTCAC
ZV_seqC_s	GAGGTRAGATCCTACTGC
ZV_seqC_a	ACCATCCATCTCAGCCTC
ZV_seqD_s	AACTCCACACTGGAACAAC
ZV_seqE_s	GAATGTCCTGGTTCTCAC
ZV_seqE_a	TAGCCTAGATCACTGTG
ZV_seqF_s	CTCAAACATAGAGCATGG
ZV_seqF_a	GTCACCATDGACCTYACTAAG
