# Supplementary material for: Potency and breadth of human primary ZIKV immune sera shows that Zika viruses cluster antigenically as a single serotype
Source: PLoS Negl Trop Dis. 2020 Apr 13;14(4):e0008006. doi: 10.1371/journal.pntd.0008006 (PMC7213746; doi:10.1371/journal.pntd.0008006)
Supplement: S2 Table — Cells highlighted in red show serum pairs that differ by P<0.05 by Bonferroni correction. (PDF) [file pntd.0008006.s002.pdf]

Supplemental Table 2. Raw P-values for pair wise comparisons of potency/breadth curves (Figure 3b). Cells highlighted in red show serum pairs that differ by P<0.05 by Bonferroni correction.

Serum	11942	11981	14278	12451	14137	11884	12462	14236	14252	14269	14276	14664
11942		0.845200	0.046900	0.968500	0.348100	0.474500	0.000014	0.0.000902	0.001700	0.001700	0.438200	0.521900
11981	0.845200		0.161300	0.930800	0.753600	0.287600	0.002200	0.001100	0.001400	0.002200	0.762500	0.449000
14278	0.046900	0.161300		0.114500	0.502200	0.415900	0.000002	0.002300	0.028400	0.021600	0.211500	0.720800
12451	0.968500	0.930800	0.114500		0.514600	0.000091	0.000091	0.000500	0.035700	0.001700	0.437900	0.638300
14137	0.348100	0.753600	0.502200	0.514600		0.826900	0.000200	0.000700	0.010700	0.005900	0.968100	0.861200
11884	0.474500	0.287600	0.415900	0.000091	0.826900		0.000055	0.002600	0.004600	0.003400	0.841000	0.988400
12462	0.000014	0.002200	0.000002	0.000091	0.000200	0.000055		0.000010	0.000001	0.255700	0.040800	0.000600
14236	0.0.000902	0.001100	0.002300	0.000500	0.000700	0.002600	0.000010		0.917100	0.255700	0.040800	0.007000
14252	0.001700	0.001400	0.028400	0.035700	0.010700	0.004600	0.000001	0.917100		0.450200	0.093500	0.014200
14269	0.001700	0.002200	0.021600	0.001700	0.005900	0.003400	0.255700	0.255700	0.450200		0.009900	0.009500
14276	0.438200	0.762500	0.211500	0.437900	0.968100	0.841000	0.040800	0.040800	0.093500	0.009900		0.854600
14664	0.521900	0.449000	0.720800	0.638300	0.861200	0.988400	0.000600	0.007000	0.014200	0.009500	0.854600	
