# Supplementary material for: Potency and breadth of human primary ZIKV immune sera shows that Zika viruses cluster antigenically as a single serotype
Source: PLoS Negl Trop Dis. 2020 Apr 13;14(4):e0008006. doi: 10.1371/journal.pntd.0008006 (PMC7213746; doi:10.1371/journal.pntd.0008006)
Supplement: S3 Table — (PDF) [file pntd.0008006.s003.pdf]

Supplemental Table 3. Summary of variable residues across ZIKV capsid (C), prM, and envelope (E) proteins.

	Capsid				Intergenic							prM										M		
**Protein position**	19	21	74	95	100	101	102	104	107	108	114	1	3	8	17	21	26	29	31	35	36	124	138	140
**Polyprotein Position**	25	27	80	101	106	107	108	110	113	114	120	123	125	130	139	143	148	151	153	157	158	246	260	262
**Consensus**	S	F	I	K	A	D	T	V	V	G	A	A	V	S	S	E	P	L	M	Y	I	R	A	A
Uganda 1947	N	L	.	R	.	.	.	I	I	.	.	.	I	.	.	K	A	.	V	H	V	K	V	V
Senegal 1984	N	L	.	R	.	.	.	I	.	.	.	.	I	.	.	K	A	.	V	H	V	K	V	V
Nigeria 1968 (A)	N	L	.	R	.	.	A	I	.	S	V	.	I	.	.	K	V	.	V	H	V	K	V	V
Nigeria 1968 (G)	N	L	.	R	.	.	A	I	.	S	V	.	I	.	.	K	V	.	V	H	V	K	V	V
Malaysia 1966	.	.	.	.	.	.	.	.	.	.	.	V	.	.	.	.	.	.	V	.	.	.	.	.
Thailand 2013	.	.	.	.	T	.	.	.	.	.	.	.	.	.	.	.	.	.	.	.	.	.	.	.
Cambodia 2010	.	.	.	.	T	.	.	.	.	.	.	V	.	N	.	.	.	M	.	.	.	.	.	.
French Polynesia 2013	.	.	.	.	.	.	.	.	.	.	.	.	.	.	N	.	.	.	.	.	.	.	.	.
Puerto Rico 2015	.	.	T	.	.	.	.	.	.	.	.	.	.	.	N	.	.	.	.	.	.	.	.	.
Brazil 2015	.	.	.	.	.	.	.	.	.	.	.	.	.	.	N	.	.	.	.	.	.	.	.	.
Colombia 2015	.	.	.	.	.	E	.	.	.	.	.	.	.	.	N	.	.	.	.	.	.	.	.	.
Mexico 2016	.	.	.	.	.	.	.	.	.	.	.	.	.	.	N	.	.	.	.	.	.	.	.	.

	E																												
**Protein position**	120	138	153	154	155	156	157	158	159	160	169	227	232	283	285	317	330	335	341	343	345	393	401	406	437	438	473	487	495
**Polyprotein Position**	410	428	443	444	445	446	447	448	449	450	459	517	522	573	575	607	620	625	631	633	635	683	691	696	727	728	763	777	785
**Consensus**	A	R	V	N	D	T	G	H	E	T	I	A	G	R	S	I	V	T	V	A	M	E	H	T	A	L	X	M	L
Uganda 1947	T	.	-	-	-	-	.	Y	.	.	V	.	.	K	F	V	.	.	I	V	.	D	.	.	V	F	V	T	M
Senegal 1984	T	.	.	.	.	.	.	.	.	.	V	.	.	.	F	V	.	.	.	.	.	D	.	.	V	F	V	T	M
Nigeria 1968 (A)	T	Q	.	.	-	-	-	-	-	-	V	S	E	.	.	V	.	R	.	.	.	D	.	I	V	F	V	T	M
Nigeria 1968 (G)	T	.	.	.	-	-	-	-	-	-	V	S	E	.	.	V	.	R	.	.	.	D	.	I	V	F	V	T	M
Malaysia 1966	.	.	.	.	.	.	.	.	.	.	.	.	.	.	.	.	.	.	.	.	.	D	Y	.	.	.	V	T	.
Thailand 2013	.	.	.	.	.	.	.	.	.	.	.	.	.	.	.	.	.	.	.	.	.	.	.	.	.	.	M	.	.
Cambodia 2010	.	.	.	.	.	.	.	.	.	.	.	.	.	.	.	.	.	.	.	.	.	.	.	.	.	.	V	.	.
French Polynesia 2013	.	.	.	.	.	.	.	.	.	.	.	.	.	.	.	.	.	.	.	.	I	.	.	.	.	.	M	.	.
Puerto Rico 2015	.	.	.	.	.	.	.	.	.	.	.	.	.	.	.	.	L	.	.	.	.	.	.	.	.	.	M	.	.
Brazil 2015	.	.	.	.	.	.	.	.	.	.	.	.	.	.	.	.	.	.	.	.	.	.	.	.	.	.	M	.	.
Colombia 2015	.	.	.	.	.	.	.	.	.	.	.	.	.	.	.	.	.	.	.	.	.	.	.	.	.	.	M	.	.
Mexico 2016	.	.	.	.	.	.	.	.	.	.	.	.	.	.	.	.	.	.	.	.	.	.	.	.	.	.	M	.	.
